# Immunohistochemical Classification and Prognostic Significance of Germinal Center-like and Memory Cell-like Subtypes of Follicular Lymphoma Using FOXP1, LMO2, CD22, and MUM1

**DOI:** 10.3390/ijms27198611

**Published:** 2026-09-26

**Authors:** Tülay Koç, Sedanur Aydın, Zeynep Öztürk, Hatice Terzi

**Affiliations:** 1 Department of Pathology, Faculty of Medicine, Sivas Cumhuriyet University, Sivas 58140, Türkiye; zeynepztrkk@gmail.com; 2 Department of Pathology, Sivas Numune Hospital, Sivas 58140, Türkiye; ksedanur@gmail.com; 3 Department of Hematology, Faculty of Medicine, Sivas Cumhuriyet University, Sivas 58140, Türkiye; dr.terzi@hotmail.com

**Keywords:** follicular lymphoma, LMO2, FOXP1, immunohistochemistry, germinal center-like subtype, memory-like subtype, prognosis, overall survival

## Abstract

Follicular lymphoma (FL) is an indolent B-cell lymphoma with considerable biological and clinical heterogeneity, highlighting the need for additional prognostic biomarkers beyond conventional clinicopathological parameters. This study aimed to evaluate the prognostic significance of an immunohistochemical classification separating FL into a germinal center-like (GC-like) phenotype, characterized by retention of germinal-center-associated features, and a memory-like (Mem-like) phenotype, reflecting a more differentiated post-germinal-center/memory B-cell state. Fifty-one patients with FL were retrospectively analyzed for clinicopathological characteristics, immunohistochemical findings, relapse, and overall survival (OS). LMO2 expression was detected in 70.6% of cases and was associated with lower mortality (19.4% vs. 60.0%, *p* = 0.008), lower relapse rates (8.3% vs. 33.3%, *p* = 0.039), and improved 5-year OS (85.3% vs. 35.0%, log-rank *p* = 0.003). FOXP1 positivity was observed in 41.2% of cases and was associated with higher mortality (57.1% vs. 13.3%, *p* = 0.002) and inferior 5-year OS (44.5% vs. 89.4%, log-rank *p* < 0.001). Mem-like patients had significantly poorer 5-year OS than GC-like patients (39.5% vs. 90.1%, log-rank *p* < 0.001). These findings suggest that LMO2 and FOXP1 expression may provide complementary prognostic information and help identify clinically distinct immunophenotypic subsets in FL.

## 1. Introduction

Follicular lymphoma (FL) is the most common indolent non-Hodgkin lymphoma and B-cell neoplasm characterized by an indolent clinical course with a median overall survival reaching 20 years with the current treatment modalities [1,2]. However, FL is a heterogeneous disease in terms of its clinical behavior and its response to immunochemotherapy (ICT), which is the standard first-line treatment [3]. Molecular cell-of-origin classification has enabled the prognostic molecular categorization of diffuse large B-cell lymphoma (DLBCL) into germinal center (GC) and activated B-cell-like (ABC) subtypes, as well as distinct genetic subgroups [4].

Unlike DLBCL, which has well-established molecular subtypes supported by multiple studies, the classification of FL remains relatively less defined. Several studies have contributed to our understanding of the heterogeneity among FL patients. Koues et al. reported that distinct subtypes can be recognized in FL based on the epigenome, with subtype 1 resembling normal centrocytes and subtype 2 resembling in vitro-activated peripheral blood B-cells [5].

Although the disease generally follows an indolent course, it is highly heterogeneous in terms of clinical behavior; while some patients remain stable for prolonged periods, others experience early progression, treatment refractoriness, or transformation to DLBCL. To explain this clinical variability, histologic grade, follicular pattern, clinical staging, and prognostic scoring systems such as FLIPI and FLIPI2 have long been used; however, these parameters do not adequately capture the biological diversity of the disease. Response to immunochemotherapy remains largely unpredictable using currently available prognostic tools based on clinicobiological parameters, such as the Follicular Lymphoma International Prognostic Index (FLIPI), FLIPI-2, and PRIMA-PI, or risk prediction models such as m7-FLIPI that combine high-risk clinical and genetic features. The impact of these tools on clinical management is limited. Therefore, there is an urgent need for precision medicine approaches that enable better risk stratification at diagnosis and the prospective selection of optimal initial therapy [2].

One study identified five genetic subtypes of FL, each associated with distinct gene mutation patterns, DNA methylation profiles, and clinical outcomes. However, that study had several limitations. The sequencing panel was designed with a relatively narrow scope, and clustering was performed exclusively on the basis of gene mutation data. In addition, information on t(14;18) translocation status was available for only a subset of the included cases, precluding its incorporation into the cluster identification process. Furthermore, the cohort used for cluster development was heterogeneous, and the associations between genetic subtypes and clinical outcomes warrant further investigation in future studies to better define the prognostic significance of these subtypes in FL, particularly in the setting of a rapidly evolving therapeutic landscape. Nevertheless, the findings of that study contributed to a deeper understanding of the molecular and clinical heterogeneity of FL and may provide a basis for the development of subtype-specific therapeutic strategies aimed at improving patient outcomes. For example, mTOR inhibitors may be effective in the AR subtype, whereas epigenetic therapies may be more suitable for the CS and GM subtypes. In contrast, patients classified within the TT subtype may derive greater benefit from chemotherapy-free therapeutic approaches [1].

Immunohistochemistry (IHC) offers a practical and cost-effective alternative for molecular subtyping using formalin-fixed paraffin-embedded (FFPE) tissue. Several IHC markers have been proposed to recapitulate gene expression profiling (GEP)-defined subtypes in FL. FOXP1 (Forkhead box P1), a transcription factor involved in B-cell maturation and post-GC differentiation, has been identified as a marker of adverse prognosis in both DLBCL and FL. LMO2 (LIM domain only 2), a transcriptional coregulator essential for early B-cell and hematopoietic development, is highly expressed in GCB-type lymphomas and correlates with favorable prognosis. CD22, a B-cell surface glycoprotein involved in B-cell receptor signaling, is broadly expressed in mature B cells including FL and may reflect GC origin. MUM1 (Multiple Myeloma Oncogene 1/IRF4), a transcription factor marking post-GC and plasma cell differentiation, has been variably expressed in FL and associated with adverse clinical behavior [6,7,8].

As GEP remains impractical for routine diagnostics, Laurent et al. developed a hierarchical IHC classifier (FLcm) using FOXP1, LMO2, CD22, and MUM1, which demonstrated significant correlation [2]. In the present study, we aimed to classify FL cases as GC-like or Mem-like subtypes using this four-marker panel and to evaluate the prognostic implications of such classification in terms of overall survival (OS) and clinical-pathological parameters.

## 2. Results

### 2.1. Clinicopathological Characteristics of the Study Cohort

A total of 51 patients diagnosed with follicular lymphoma between March 2015 and July 2025 were included in the study. The mean age at diagnosis was 56.9 ± 11.6 years, with a median age of 58 years (range, 30–76 years). Twenty-six patients (51.0%) were male and 25 (49.0%) were female.

The most common biopsy/localization sites were the inguinal region (n = 17, 33.3%) and axillary region (n = 14, 27.5%). A follicular growth pattern was observed in 43 cases (84.3%), whereas 8 cases (15.7%) showed a diffuse pattern. Histologically, 21 cases (41.2%) were grade 1, 15 (29.4%) were grade 2, 7 (13.7%) were grade 3A, and 8 (15.7%) were grade 3B.

Most patients presented with advanced-stage disease; 46 patients (90.2%) had stage IV disease. Elevated serum LDH levels were observed in 21 patients (41.2%), and bone marrow involvement was present in 19 (37.3%). The median number of involved lymph nodes was 4 (range, 0–8). According to FLIPI categories, 17 patients (33.3%) were classified as low risk, 15 (29.4%) as intermediate risk, and 19 (37.3%) as high risk. During follow-up, relapse was observed in 8 patients (15.7%), whereas 16 patients (31.4%) died.

Thirty-nine patients (76.5%) received chemotherapy, whereas 12 (23.5%) did not. During follow-up, relapse was recorded in 8 patients (15.7%). Because relapse represented a post-baseline event and exact relapse dates were unavailable, it was not included as a baseline prognostic variable in the survival models. The clinicopathological characteristics of the cohort are summarized in Table 1.

### 2.2. Immunophenotypic Classification Using the FLcm Algorithm and Comparison of Baseline Characteristics

LMO2 expression was positive in 36 patients (70.6%) and negative in 15 (29.4%). FOXP1 expression was positive in 21 patients (41.2%) and negative in 30 (58.8%).

In six cases, the difference between FOXP1 and LMO2 expression was <20%, precluding direct classification at the first step of the FLcm algorithm. These cases were therefore subjected to CD22 evaluation. Three cases showed CD22 expression in >80% of tumor cells and were assigned to the GC-like subtype, whereas two cases with CD22 expression <20% were classified as Mem-like. One case exhibited intermediate CD22 expression (40%) and was subsequently evaluated for MUM1. Because this case demonstrated diffuse MUM1 positivity, it was ultimately classified as Mem-like. Accordingly, the hierarchical use of CD22 and MUM1 resolved all six initially indeterminate cases and allowed complete assignment of the cohort into GC-like and Mem-like subtypes.

According to the combined immunophenotypic classification, 19 patients (37.3%) were classified as Mem-like subtype and 32 (62.7%) as GC-like subtype. Representative immunohistochemical staining patterns are shown in Figure 1, Figure 2 and Figure 3.

Baseline clinicopathological characteristics were generally comparable between mem-like and GC-like. Mean age was 56.1 ± 10.8 years in mem-like and 57.4 ± 12.2 years in GC-like (*p* = 0.695), and sex distribution was similar between the two groups (*p* = 0.776).

No statistically significant differences were observed between mem-like and GC-like with respect to growth pattern, histological grade, clinical stage, LDH level, bone marrow involvement, or receipt of chemotherapy. The median number of involved lymph nodes was 5 in mem-like and 3.5 in GC-like, but this difference was not statistically significant (*p* = 0.296).

Although the proportion of patients in FLIPI high risk group was numerically higher in mem-like than in GC-like (52.6% vs. 28.1%), the overall distribution of FLIPI categories did not differ significantly between the two groups (*p* = 0.184). These findings indicate that the two immunophenotypic classes were broadly comparable with respect to the principal baseline clinicopathological variables assessed. Baseline characteristics according to immunophenotypic subtype are shown in Table 2.

### 2.3. Overall Survival in the Entire Cohort

At the end of follow-up, 16 patients (31.4%) had died and 35 (68.6%) were alive and censored at their last follow-up date. Median follow-up, estimated using the reverse Kaplan–Meier method, was 65.1 months.

The estimated overall survival rates were 90.2% at 1 year (95% CI, 78.0–95.8), 72.8% at 3 years (95% CI, 57.6–83.3), and 69.5% at 5 years (95% CI, 53.5–80.9). Eighteen patients remained at risk at 5 years. Median OS was not reached for the overall cohort because the Kaplan–Meier survival curve did not fall below 50%. Overall survival estimates are presented in Table 3.

A previously calculated 10-year OS estimate was not included in the principal analysis because only one patient remained at risk at 10 years, making this long-term estimate insufficiently robust for interpretation.

### 2.4. Overall Survival According to Clinicopathological Variables

No statistically significant differences in OS were observed according to age, sex, histological grade, clinical stage, LDH level, or bone marrow involvement.

The 5-year OS was 75.3% in patients aged ≤ 60 years and 57.9% in those aged >60 years (log-rank *p* = 0.305). Five-year OS rates were 68.1% in women and 68.8% in men (*p* = 0.955). Histological grade was not associated with OS, with 5-year OS rates of 68.5% in grade 1–2 disease and 72.7% in grade ≥ 3 disease (*p* = 0.768).

Clinical stage was not significantly associated with OS (*p* = 0.089), although this comparison should be interpreted cautiously because only five patients had stage I–III disease. Patients with elevated LDH had a numerically lower 5-year OS than those with normal or low LDH levels (55.3% vs. 79.0%), but the difference was not statistically significant (*p* = 0.317). Bone marrow involvement was likewise not significantly associated with OS (*p* = 0.289).

In contrast, FLIPI category was significantly associated with survival. The 5-year OS rates were 88.2% for FLIPI category 1, 79.4% for category 2, and 37.3% for category 3 (log-rank *p* = 0.021). Median OS was 34.1 months in FLIPI category 3, whereas it was not reached in categories 1 and 2. Kaplan–Meier survival analyses are summarized in Table 4.

### 2.5. Prognostic Significance of LMO2 and FOXP1 Expression

LMO2 expression was significantly associated with improved OS. Nine of 15 LMO2-negative patients (60.0%) died during follow-up compared with 7 of 36 LMO2-positive patients (19.4%). The estimated 5-year OS was 35.0% in the LMO2-negative group (95% CI, 10.9–60.8) and 85.3% in the LMO2-positive group (95% CI, 68.2–93.6). Median OS was 34.1 months in LMO2-negative patients, whereas it was not reached in LMO2-positive patients. The difference between Kaplan–Meier survival curves was statistically significant (log-rank *p* = 0.003).

FOXP1 expression showed the opposite association. Twelve of 21 FOXP1-positive patients (57.1%) died, compared with only 4 of 30 FOXP1-negative patients (13.3%). The estimated 5-year OS was 44.5% in FOXP1-positive patients (95% CI, 21.7–65.1) and 89.4% in FOXP1-negative patients (95% CI, 70.6–96.5). Median OS was 47.7 months in the FOXP1-positive group and was not reached in the FOXP1-negative group. The survival difference was highly significant (log-rank *p* < 0.001). Kaplan–Meier survival analyses are summarized in Table 4.

### 2.6. Prognostic Significance of the Combined Immunophenotypic Classification

The combined immunophenotypic classification showed one of the strongest associations with OS. Twelve of 19 patients in Class 1 (63.2%) died during follow-up, compared with only 4 of 32 patients in Class 2 (12.5%).

The estimated 5-year OS was 39.5% in Class 1 (95% CI, 17.2–61.2) and 90.1% in Class 2 (95% CI, 72.4–96.7). Median OS was 34.1 months in Class 1, whereas it was not reached in Class 2. Kaplan–Meier survival curves demonstrated a highly significant separation between the two immunophenotypic classes (log-rank *p* < 0.001). Kaplan–Meier survival analyses are summarized in Table 4.

### 2.7. Univariable Cox Regression Analysis

In univariable Cox proportional hazards analysis, age, sex, histological grade, clinical stage, LDH level, and bone marrow involvement were not significantly associated with the hazard of death.

Each one-category increase in FLIPI was associated with a 2.25-fold increase in the hazard of death (HR = 2.25, 95% CI, 1.15–4.41; *p* = 0.018).

LMO2 positivity was associated with a significantly lower hazard of death (HR = 0.25, 95% CI, 0.09–0.68; *p* = 0.006), whereas FOXP1 positivity was associated with a 5.61-fold higher hazard of death (HR = 5.61, 95% CI, 1.78–17.68; *p* = 0.003).

Immunophenotypic class showed the strongest univariable association with outcome. Patients in Class 1 had a 6.64-fold higher hazard of death than those in Class 2 (HR = 6.64, 95% CI, 2.12–20.82; *p* = 0.001). Cox regression results are presented in Table 5.

### 2.8. Multivariable Cox Regression Analysis

Because only 16 deaths occurred in the cohort, the multivariable model was deliberately restricted to minimize overfitting and unstable coefficient estimates. In addition, LMO2, FOXP1, and the combined immunophenotypic class were not entered simultaneously because of their biological and classificatory interdependence. Therefore, the primary multivariable Cox model included only immunophenotypic class and FLIPI.

After adjustment for FLIPI, Class 1 remained significantly associated with inferior OS, with an approximately five-fold higher hazard of death compared with Class 2 (adjusted HR = 5.08, 95% CI, 1.53–16.80; *p* = 0.008). In the same model, FLIPI was no longer statistically significant (adjusted HR = 1.58, 95% CI, 0.81–3.10; *p* = 0.181).

Evaluation of Schoenfeld residuals for the primary two-variable Cox model showed no clear evidence of time-dependent effects, indicating no major violation of the proportional hazards assumption.

Bootstrap validation supported the robustness of the primary multivariable Cox proportional hazards model. Among 2000 nonparametric bootstrap resamples, 1996 yielded valid model estimates. The association between Class 1 immunophenotype and poorer overall survival remained stable, with a bootstrap median HR of 5.59 and a bias-corrected and accelerated (BCa) 95% CI of 1.54–17.54. In contrast, FLIPI did not demonstrate an independently robust association with overall survival in the bootstrap analysis, with a bootstrap median HR of 1.58 and a BCa 95% CI of 0.77–3.88. Overall, these findings supported the internal stability of the prognostic effect observed for the immunophenotypic classification. Cox regression results are presented in Table 5.

## 3. Discussion

In this retrospective single-center study of 51 patients with classic follicular lymphoma, we applied the four-marker immunohistochemical classifier (FLcm) proposed by Laurent et al. to a routine diagnostic cohort and evaluated its prognostic relevance [2]. All cases could be assigned to one of the two predefined classes: 32 cases (62.7%) were GC-like and 19 (37.3%) were Mem-like. This distribution is consistent with the germinal center origin of the majority of FL cases and with the proportion of Mem-like tumors reported in the original gene expression-based and immunohistochemical series [2,4]. Importantly, classification was achieved on FFPE tissue with commercially available antibodies and an automated staining platform, supporting the feasibility of the approach in routine pathology practice.

The most striking finding of the present study is the magnitude of the survival difference between the two immunophenotypic subtypes. Mortality during follow-up was 63.2% in Mem-like patients versus 12.5% in GC-like patients (*p* < 0.001), the median OS was 34.1 months in the Mem-like group but was not reached in the GC-like group, and the estimated 5-year OS was 39.5% versus 90.1% (log-rank *p* < 0.001). In univariable Cox regression, GC-like classification was associated with an 84% reduction in the hazard of death (HR = 0.16, 95% CI 0.05–0.51).

Although relapse was numerically more frequent among Mem-like patients (26.3% vs. 9.4%), this difference did not reach statistical significance, most likely because of the small number of relapse events (n = 8) in the cohort. Taken together, these results confirm in an independent, real-world population that the GC-like/Mem-like dichotomy captures clinically meaningful biological heterogeneity in FL, in analogy to the cell-of-origin classification of DLBCL [4].

Importantly, the prognostic significance of the immunophenotypic classification persisted after adjustment for FLIPI. In the parsimonious multivariable Cox model, the Mem-like subtype was associated with an approximately five-fold higher hazard of death compared with the GC-like subtype (adjusted HR = 5.08, 95% CI 1.53–16.80; *p* = 0.008), whereas FLIPI was no longer statistically significant in the same model (adjusted HR = 1.58, 95% CI 0.81–3.10; *p* = 0.181). These findings suggest that the FLcm-defined immunophenotypic classification may capture prognostic information not fully reflected by conventional clinical risk stratification. Nevertheless, given the limited number of deaths, these multivariable estimates should be interpreted cautiously and validated in larger independent cohorts.

LMO2 and FOXP1, the two markers that form the first step of the FLcm algorithm, each showed strong and opposite prognostic associations when analyzed individually. LMO2 was expressed in 70.6% of cases and was associated with lower mortality (19.4% vs. 60.0%), fewer relapses (8.3% vs. 33.3%), a higher 5-year OS (85.3% vs. 35.0%) and a hazard ratio for death of 0.25. LMO2 is a transcriptional coregulator essential for early hematopoietic and B-cell development, is one of the principal drivers of the GC B-cell gene expression signature, and has previously been shown to predict survival in DLBCL treated with anthracycline-based chemotherapy with or without rituximab [6]. Our data extend this favorable prognostic role of LMO2 protein expression to FL and are in line with the observation that LMO2 is among the most discriminative genes in the FL20 signature underlying the GC-like/Mem-like classification [2].

FOXP1, in contrast, was positive in 41.2% of cases and was associated with substantially higher mortality (57.1% vs. 13.3%), a median OS of 47.7 months and a 5-year OS of 44.5% compared with 89.4% in FOXP1-negative patients; FOXP1 positivity conferred an approximately 5.6-fold increase in the hazard of death (HR = 5.61, 95% CI 1.78–17.68). Relapse was also more frequent in FOXP1-positive patients (28.6% vs. 6.7%), although this difference was of borderline significance (*p* = 0.052). FOXP1 is a forkhead transcription factor that represses the germinal center program, promotes post-GC and plasmacytic differentiation, and has long been recognized as an adverse prognostic marker in DLBCL [8]. In FL, Mottok et al. demonstrated that high FOXP1 expression in patients treated with rituximab and chemotherapy is associated with inferior failure-free survival, particularly in tumors with concurrent TP53 mutation and IRF4 expression [9]. Our findings independently corroborate the adverse prognostic impact of FOXP1 protein expression in FL and, notably, show that its effect on OS in our cohort was at least as strong as that of LMO2 loss.

It is noteworthy that the combined FLcm-based classification yielded the most pronounced separation of survival curves, with 5-year OS rates of 90.1% in the GC-like group and 39.5% in the Mem-like group. This supports the rationale of the hierarchical FLcm algorithm, in which LMO2 and FOXP1 are read jointly and cases with a discordant or narrow gap between the two markers are resolved by CD22 and, if necessary, MUM1/IRF4. CD22 is a B-cell receptor-associated surface glycoprotein that is retained in GC-derived B cells and is therefore informative as a second-step GC marker, whereas MUM1/IRF4 marks post-GC exit and plasma cell commitment [7]. The prognostic relevance of MUM1/IRF4 in FL is increasingly recognized: gain of 6p encompassing the IRF4 locus has been associated with adverse outcome, and, although rare in conventional cFL, IRF4 rearrangements have been described that produce atypical immunophenotypes with strong MUM1 expression [10,11]. The inclusion of these two markers as tie-breakers therefore allows a biologically coherent assignment even in immunophenotypically ambiguous cases, which is essential for a classifier intended for routine use.

Our cohort was characterized by a high proportion of advanced-stage disease (90.2% stage IV) and a substantial fraction of FLIPI high-risk patients (37.3%), which likely contributed to the relatively high overall mortality (31.4%) and a 5-year OS of 69.5% for the entire cohort, values lower than those reported in contemporary clinical trial populations. This composition, however, also underlines the clinical relevance of our observations: even within a predominantly advanced-stage population, immunophenotypic subtyping identified a subgroup (Mem-like) with a markedly inferior outcome and, conversely, a large subgroup (GC-like) with an excellent long-term survival. Current prognostic tools, including FLIPI, FLIPI2, PRIMA-PI and m7-FLIPI, rely on clinical and, in the latter case, genetic parameters and have limited ability to predict response to immunochemotherapy [12,13]. The validation of POD24 as a robust surrogate of poor survival across 5225 patients and 13 clinical trials has emphasized the need to identify patients at risk of early progression at the time of diagnosis rather than retrospectively [14]. Although POD24 could not be directly evaluated in our cohort because exact treatment initiation and progression dates were not consistently available, the markedly inferior OS observed in the Mem-like subgroup raises the hypothesis that this phenotype may identify patients with biologically more aggressive disease. This potential association requires validation in cohorts with prospectively documented treatment and progression data. Together with the emerging genetic subtypes of FL and their proposed subtype-specific therapeutic implications [1], immunophenotypic classification may thus contribute to a more individualized management of FL.

Several limitations of this study should be acknowledged. First, the sample size was modest and the analysis was retrospective and single-center, which limits statistical power, particularly for relapse-related endpoints, and may introduce selection bias. Second, although the immunophenotypic classification remained significantly associated with OS after adjustment for FLIPI, the limited number of death events restricted the number of covariates that could be included in the multivariable model. Therefore, the independence of this association from additional factors such as histological grade, treatment regimen, and other molecular variables requires validation in larger cohorts. Third, treatment was not uniform across the study period, and the prognostic value of the classifier may differ between rituximab-based, obinutuzumab-based and chemotherapy-free regimens. Fourth, the FLcm classification was not validated against gene expression profiling in our cohort, and cut-off values for the individual markers were adopted from the original publication without local recalibration. In addition, although all immunohistochemical assessments were reviewed by two pathologists, individual observer-level scores were not retained, precluding a formal assessment of interobserver reproducibility using Cohen’s κ or ICC. Future validation studies should specifically evaluate the reproducibility of FLcm scoring across observers and institutions. Finally, histologic transformation and progression-free survival were not systematically evaluated, and the relatively short median follow-up may underestimate late events in the GC-like group. Prospective, multicenter studies with larger cohorts, uniform treatment and integration of genetic data are needed to establish whether the GC-like/Mem-like immunophenotypic classification provides prognostic information independent of, and additive to, existing indices.

## 4. Materials and Methods

### 4.1. Study Design and Patient Selection

This retrospective study included 51 adult patients with histopathologically confirmed cFL diagnosed at our institution between March 2015 and July 2025. Inclusion criteria were: (i) diagnosis of cFL according to WHO-HAEM5 criteria; (ii) availability of adequate FFPE tissue for IHC; and (iii) complete clinical and follow-up data. Patients with documented histologic transformation at diagnosis, those with insufficient tissue, and pediatric cases were excluded. The study was approved by the institutional ethics committee (Approval No: 2025-12/61).

### 4.2. Histopathological Evaluation

All hematoxylin–eosin (H&E) stained sections were reviewed by two experienced pathologists blinded to clinical outcomes. Histological classification and grading were performed according to WHO-HAEM5 criteria; tumor growth pattern (follicular, follicular-diffuse) was recorded.

### 4.3. Immunohistochemical Staining

IHC staining was performed on 4 μm-thick FFPE sections using an automated immunostainer (Ventana BenchMark ULTRA; Roche Diagnostics, Basel, Switzerland). The following primary antibodies were used: anti-FOXP1 (clone SP133, Cellmarq), anti-LMO2 (clone SP51, Genlab), anti-CD22 (clone SP104, Cellmarq), and anti-MUM1 (clone Ep190, Roche). Appropriate positive and negative controls were included in each run.

### 4.4. IHC Scoring and FLcm Classification

Cases were classified using the hierarchical FLcm algorithm described.

Step 1: Both FOXP1 and LMO2 are evaluated simultaneously. If there is a clear difference (gap ≥ 20%) between the two stainings, the case is classified as MEM-like (FOXP1 > 50%, LMO2 < 50%) or GC-like (FOXP1 < 50%, LMO2 > 50%).

Step 2: If the gap is <20%, CD22 is added; strong CD22 (>80%) = GC-like; negative CD22 (<20%) = MEM-like.

Step 3: In cases of intermediate CD22 (20–80%), MUM1 is assessed; MUM1 > 15% = MEM-like; MUM1 ≤ 15% = GC-like.

### 4.5. Clinical Data and Follow-Up

Clinical parameters included age, sex, Ann Arbor stage, serum LDH, number of involved nodal sites, bone marrow involvement. The FLIPI was calculated for all patients. OS was defined as the interval from diagnosis to death from any cause or last follow-up.

### 4.6. Statistical Analysis

Statistical analyses were performed using SPSS v23.0 (IBM Corp., Armonk, NY, USA). Categorical variables were compared using the Chi-square (χ^2^) or Fisher’s exact test. Continuous variables are expressed as mean ± standard deviation (SD) and were compared using Student’s *t*-test or the Mann–Whitney U test. OS was analyzed using the Kaplan–Meier method and compared with the log-rank test. Given the limited number of death events, multivariable Cox proportional hazards regression was restricted to a parsimonious model including immunophenotypic class and FLIPI to minimize overfitting. LMO2, FOXP1, and immunophenotypic class were not entered simultaneously because of their biological and classificatory interdependence. The proportional hazards assumption was assessed using Schoenfeld residuals. A two-sided *p* < 0.05 was considered statistically significant.

## 5. Conclusions

In this retrospective series of 51 patients with classic follicular lymphoma, the four-marker immunohistochemical classifier based on FOXP1, LMO2, CD22 and MUM1 allowed unambiguous assignment of every case to a GC-like or Mem-like subtype using routine FFPE tissue and standard automated staining. The two subtypes differed markedly in outcome, with estimated 5-year OS rates of 39.5% for Mem-like and 90.1% for GC-like patients. Moreover, Mem-like classification remained independently associated with inferior OS after adjustment for FLIPI (adjusted HR = 5.08, 95% CI 1.53–16.80; *p* = 0.008). LMO2 expression and FOXP1 expression each carried strong and opposite prognostic significance on their own, whereas their combined interpretation within the FLcm algorithm provided the clearest separation of survival curves. These findings indicate that immunophenotypic subtyping is a feasible, inexpensive and reproducible tool that captures biologically and clinically relevant heterogeneity in FL and may help identify, at the time of diagnosis, patients at risk of early death who could benefit from closer follow-up or alternative treatment strategies. Prospective multicenter studies with uniform treatment and integration of genetic data are needed to confirm the independence of this classification from established prognostic indices and to define its role in treatment selection.

## Figures and Tables

**Figure 1:**
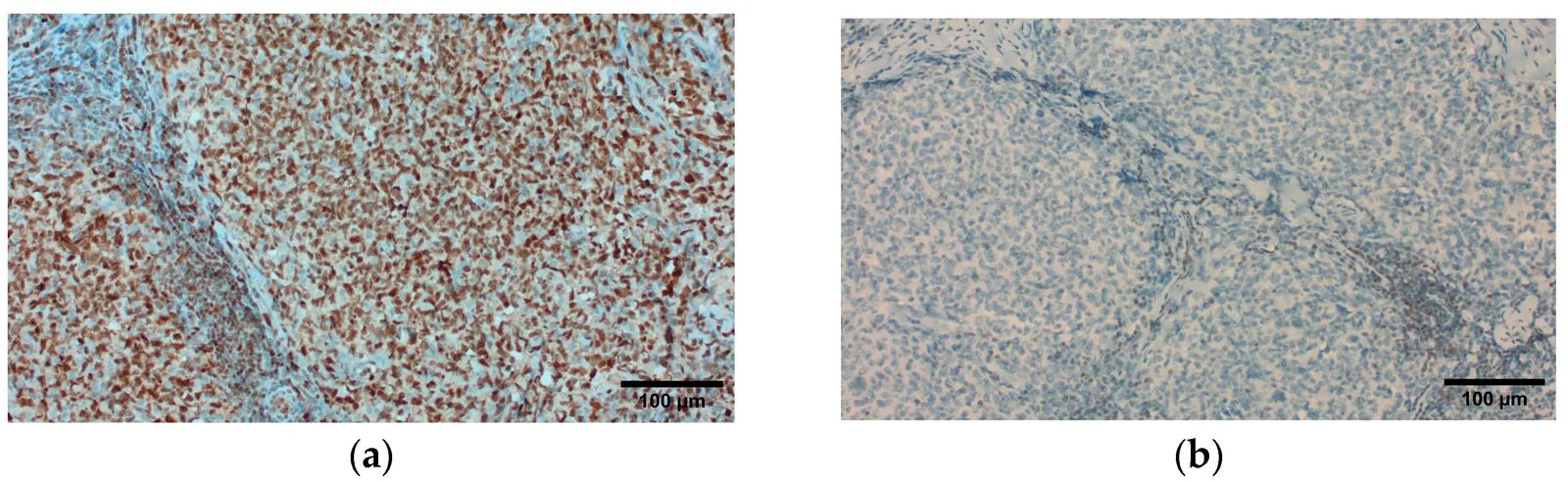
GC-like subtype: (**a**) LMO2 positive in approximately 90% of tumor cells (×200); (**b**) FOXP1 negative (×200).

**Figure 2:**
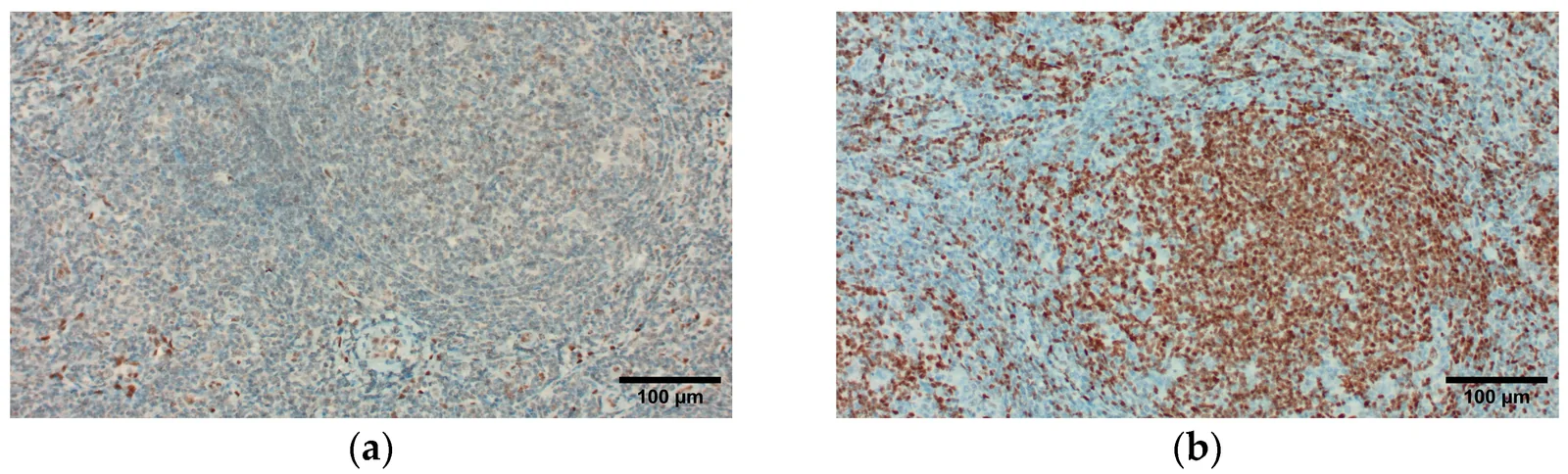
Mem-like subtype: (**a**) LMO2 negative (×200); (**b**) FOXP1 positive in approximately 80% of tumor cells (×200).

**Figure 3:**
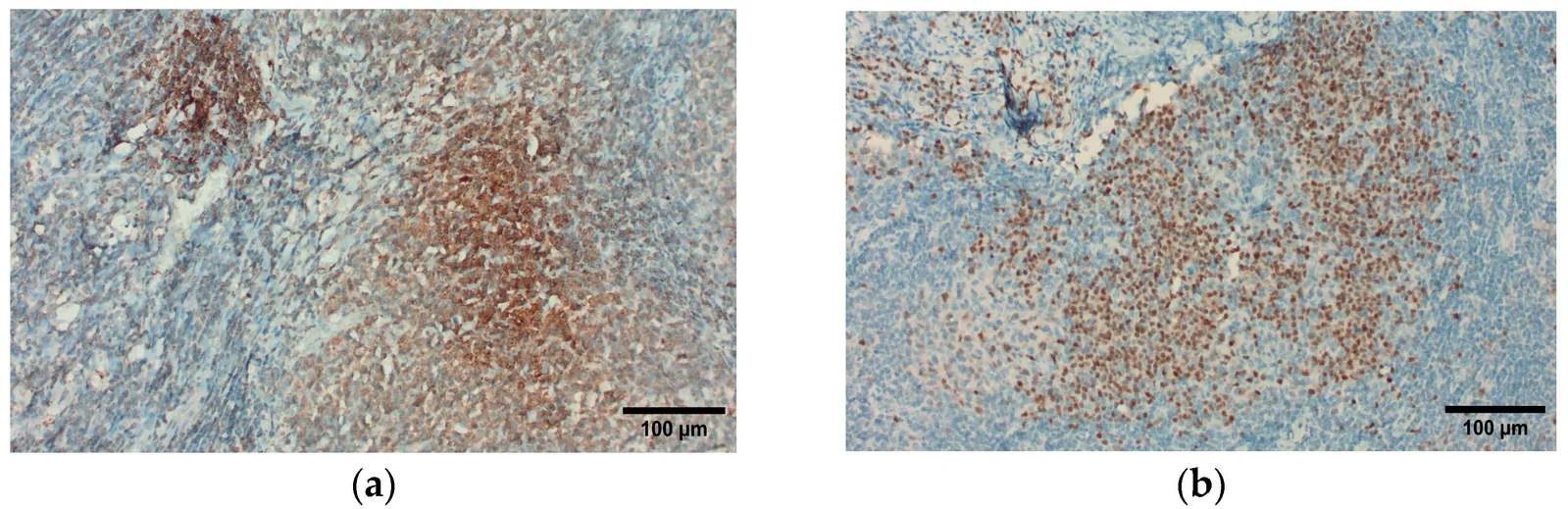
(**a**) CD22 positive (×200), GC-like subtype; (**b**) MUM-1 positive (×200), Mem-like subtype.

**Table 1:** Demographic, clinicopathological, treatment, and immunophenotypic characteristics of the study cohort.

Characteristic	Category	Value
Total patients		51
Age, years	Mean ± SD	56.9 ± 11.6
	Median (range)	58 (30–76)
Sex	Female	25 (49.0%)
	Male	26 (51.0%)
Localization	Inguinal	17 (33.3%)
	Axillary	14 (27.5%)
	Cervical	5 (9.8%)
	Abdominal	4 (7.8%)
	Other	11 (21.6%)
Growth pattern	Follicular	43 (84.3%)
	Diffuse	8 (15.7%)
Histological grade	Grade 1	21 (41.2%)
	Grade 2	15 (29.4%)
	Grade 3A	7 (13.7%)
	Grade 3B	8 (15.7%)
Clinical stage	I	2 (3.9%)
	II	1 (2.0%)
	III	2 (3.9%)
	IV	46 (90.2%)
LDH level	Normal/low	30 (58.8%)
	Elevated	21 (41.2%)
Involved lymph nodes, n	Mean ± SD	3.96 ± 2.23
	Median (range)	4 (0–8)
Bone marrow involvement	Absent	32 (62.7%)
	Present	19 (37.3%)
FLIPI category	Low risk	17 (33.3%)
	Intermediate risk	15 (29.4%)
	High risk	19 (37.3%)
Chemotherapy	No	12 (23.5%)
	Yes	39 (76.5%)
Relapse during follow-up	No	43 (84.3%)
	Yes	8 (15.7%)
LMO2 expression	Negative	15 (29.4%)
	Positive	36 (70.6%)
FOXP1 expression	Negative	30 (58.8%)
	Positive	21 (41.2%)
Immunophenotypic class	Mem-like	19 (37.3%)
	GC-like	32 (62.7%)

Data are presented as n (%), mean ± standard deviation (SD), or median (range), as appropriate. LDH, lactate dehydrogenase; FLIPI, Follicular Lymphoma International Prognostic Index.

**Table 2:** Comparison of baseline clinicopathological characteristics according to immunophenotypic class.

Characteristic	Mem-like (n = 19)	GC-like (n = 32)	*p*
Age, years, mean ± SD	56.1 ± 10.8	57.4 ± 12.2	0.695
Sex			0.776
Female	10 (52.6%)	15 (46.9%)	
Male	9 (47.4%)	17 (53.1%)	
Growth pattern			0.694
Follicular	17 (89.5%)	26 (81.3%)	
Diffuse	2 (10.5%)	6 (18.8%)	
Histological grade			1.000
Grade 1–2	13 (68.4%)	23 (71.9%)	
Grade 3	6 (31.6%)	9 (28.1%)	
Clinical stage			0.348
I–III	3 (15.8%)	2 (6.3%)	
IV	16 (84.2%)	30 (93.8%)	
LDH level			0.247
Normal/low	9 (47.4%)	21 (65.6%)	
Elevated	10 (52.6%)	11 (34.4%)	
Involved lymph nodes, median (range)	5 (1–8)	3.5 (0–7)	0.296
Bone marrow involvement			1.000
Absent	12 (63.2%)	20 (62.5%)	
Present	7 (36.8%)	12 (37.5%)	
FLIPI category			0.184
low	4 (21.1%)	13 (40.6%)	
intermediate	5 (26.3%)	10 (31.3%)	
high	10 (52.6%)	9 (28.1%)	
Chemotherapy			0.743
No	5 (26.3%)	7 (21.9%)	
Yes	14 (73.7%)	25 (78.1%)	

Data are presented as n (%) unless otherwise indicated. Continuous age was compared using Welch’s t-test; involved lymph node counts were compared using the Mann–Whitney U test. Fisher’s exact test was used for 2 × 2 categorical comparisons, and the chi-square test was used for FLIPI categories.

**Table 3:** Overall survival estimates for the study cohort.

Time Point	OS Estimate, %	95% CI, %	Patients at Risk, n
1 year	90.2	78.0–95.8	46
3 years	72.8	57.6–83.3	30
5 years	69.5	53.5–80.9	18

OS, overall survival; CI, confidence interval. OS was calculated from the date of diagnosis to death from any cause or last follow-up. Patients alive at the last follow-up were censored. Survival probabilities were estimated using the Kaplan–Meier method with Greenwood-based 95% confidence intervals. Median follow-up was estimated using the reverse Kaplan–Meier method. The 10-year OS estimate was not included in the principal table because only one patient remained at risk at 10 years.

**Table 4:** Kaplan–Meier analysis of overall survival according to clinicopathological and immunophenotypic characteristics.

Variable	Category	n	Deaths, n (%)	5-Year OS, % (95% CI)	Log-Rank *p*
Age	≤60 years	29	8 (27.6)	75.3 (54.9–87.4)	0.305
	>60 years	22	8 (36.4)	57.9 (27.9–79.2)	
Sex	Female	25	8 (32.0)	68.1 (44.0–83.6)	0.955
	Male	26	8 (30.8)	68.8 (43.5–84.5)	
Histological grade	Grade 1–2	36	11 (30.6)	68.5 (48.8–81.9)	0.768
	Grade ≥ 3	15	5 (33.3)	72.7 (42.5–88.8)	
Clinical stage	I–III	5	3 (60.0)	40.0 (5.2–75.3)	0.089
	IV	46	13 (28.3)	72.6 (55.4–84.1)	
LDH level	Normal/low	30	8 (26.7)	79.0 (58.9–90.0)	0.317
	Elevated	21	8 (38.1)	55.3 (28.3–75.7)	
Bone marrow involvement	Absent	32	12 (37.5)	65.6 (44.6–80.3)	0.289
	Present	19	4 (21.1)	75.8 (47.4–90.2)	
FLIPI category	1	17	3 (17.6)	88.2 (60.6–96.9)	0.021
	2	15	3 (20.0)	79.4 (48.8–92.9)	
	3	19	10 (52.6)	37.3 (11.9–63.3)	
LMO2 expression	Negative	15	9 (60.0)	35.0 (10.9–60.8)	0.003
	Positive	36	7 (19.4)	85.3 (68.2–93.6)	
FOXP1 expression	Negative	30	4 (13.3)	89.4 (70.6–96.5)	<0.001
	Positive	21	12 (57.1)	44.5 (21.7–65.1)	
Immunophenotypic class	Mem-like	19	12 (63.2)	39.5 (17.2–61.2)	<0.001
	GC-like	32	4 (12.5)	90.1 (72.4–96.7)	

OS, overall survival; CI, confidence interval; LDH, lactate dehydrogenase; FLIPI, Follicular Lymphoma International Prognostic Index. Survival probabilities were estimated using the Kaplan–Meier method with Greenwood-based 95% confidence intervals and were compared using the log-rank test. Histological grade was grouped as grade 1–2 versus grade 3 for survival analyses. Relapse was not included because it occurred after study entry and exact relapse dates were unavailable.

**Table 5:** Univariable and multivariable Cox proportional hazards regression analyses for overall survival.

Variable	Comparison/Unit	Univariable HR	95% CI	*p*
Age	Per 1-year increase	1.02	0.97–1.06	0.517
Sex	Male vs. female	1.03	0.38–2.81	0.955
Histological grade	3 vs. 1–2	1.17	0.41–3.39	0.768
Clinical stage	IV vs. I–III	0.35	0.10–1.24	0.104
LDH level	Elevated vs. normal/low	1.65	0.61–4.44	0.322
Bone marrow involvement	Present vs. absent	0.55	0.18–1.70	0.297
FLIPI	Per category increase	2.25	1.15–4.41	0.018
LMO2 expression	Positive vs. negative	0.25	0.09–0.68	0.006
FOXP1 expression	Positive vs. negative	5.61	1.78–17.68	0.003
Immunophenotypic class	Mem-like vs. GC-like	6.64	2.12–20.82	0.001

HR, hazard ratio; CI, confidence interval; LDH, lactate dehydrogenase; FLIPI, Follicular Lymphoma International Prognostic Index. The primary multivariable model included immunophenotypic class and FLIPI. The number of variables was deliberately restricted because only 16 deaths occurred, thereby reducing the risk of model overfitting. Relapse was excluded from Cox regression because it was a post-baseline event and exact relapse dates were unavailable. LMO2, FOXP1, and immunophenotypic class were not entered simultaneously because of their biological and classificatory interdependence.

## Data Availability

The data presented in this study are available from the corresponding author upon reasonable request. The data are not publicly available due to privacy and ethical restrictions.

## Abbreviations

ABC	Activated B-cell-like
cFL	Classic follicular lymphoma
CI	Confidence interval
DLBCL	Diffuse large B-cell lymphoma
FFPE	Formalin-fixed, paraffin-embedded
FL	Follicular lymphoma
FLcm	Follicular lymphoma cell-of-origin molecular classifier
FLIPI	Follicular Lymphoma International Prognostic Index
FLIPI2	Follicular Lymphoma International Prognostic Index 2
FOXP1	Forkhead box P1
GC	Germinal center
GCB	Germinal center B-cell-like
GEP	Gene expression profiling
H&E	Hematoxylin and eosin
HR	Hazard ratio
ICT	Immunochemotherapy
IHC	Immunohistochemistry
IRF4	Interferon regulatory factor 4
LDH	Lactate dehydrogenase
LMO2	LIM domain only 2
Mem-like	Memory cell-like
MUM1	Multiple myeloma oncogene 1
OS	Overall survival
POD24	Progression of disease within 24 months
PRIMA-PI	PRIMA Prognostic Index
SD	Standard deviation
WHO-HAEM5	World Health Organization Classification of Haematolymphoid Tumours, 5th edition
